# The Chloroplast Reactive Oxygen Species-Redox System in Plant Immunity and Disease

**DOI:** 10.3389/fpls.2020.572686

**Published:** 2020-11-12

**Authors:** Elżbieta Kuźniak, Tomasz Kopczewski

**Affiliations:** Department of Plant Physiology and Biochemistry, Faculty of Biology and Environmental Protection, University of Lodz, Lodz, Poland

**Keywords:** chloroplasts, defense response, pathogens, redox signaling, stress hormones

## Abstract

Pathogen infections limit plant growth and productivity, thus contributing to crop losses. As the site of photosynthesis, the chloroplast is vital for plant productivity. This organelle, communicating with other cellular compartments challenged by infection (e.g., apoplast, mitochondria, and peroxisomes), is also a key battlefield in the plant–pathogen interaction. Here, we focus on the relation between reactive oxygen species (ROS)—redox signaling, photosynthesis which is governed by redox control, and biotic stress response. We also discuss the pathogen strategies to weaken the chloroplast-mediated defense responses and to promote pathogenesis. As in the next decades crop yield increase may depend on the improvement of photosynthetic efficiency, a comprehensive understanding of the integration between photosynthesis and plant immunity is required to meet the future food demand.

## The Implication of Chloroplasts in Plant Defense

Plant diseases significantly reduce the yield of agricultural production worldwide (Nelson, 2020). In plants, there is an antagonistic relationship between immunity and growth, known as the growth—immunity trade-off. Immune responses temporarily suppress plant growth and *vice versa*—intense growth can hinder the defense reactions. A likely mechanism of this trade-off is the diversion of photosynthesis-derived energy and metabolites to the defense-related pathways instead of growth. The recognition of pathogen results in downregulation of growth mediated by phytohormones and upregulation of defense-related genes (Karasov et al., 2017). In *Arabidopsis*, constitutive accumulation of salicylic acid (SA) correlates with increased resistance to *Peronospora parasitica* but negatively affects growth (Mauch et al., 2001). Other studies also indicate that SA, a plant defense hormone, contributes to the homeostasis of plant growth and immunity (Ding and Ding, 2020).

Chloroplasts are not only vital for plant productivity, but they are also active sensors of the environment integrating the cellular response to stress. They significantly participate in the generation of ROS and NO, play a central role in redox homeostasis and retrograde signaling regulating nuclear gene expression (Foyer, 2018; Dietz et al., 2019). Their role in immunity is supported by observations that plant resistance to pathogens differ between light and dark, and light and functional chloroplasts are needed for defense responses (Roden and Ingle, 2009; Kuźniak et al., 2010).

Chloroplasts are also involved in stress hormone signaling by providing biosynthetic precursors for SA, jasmonic acid (JA), abscisic acid (ABA), and ethylene (ET) (Baier and Dietz, 2005; Lu and Yao, 2018).

As a signaling hub for regulating plant stress responses, chloroplasts are also targets for pathogen effectors and phytotoxins to suppress host defense, which makes them a key battlefield in plant–pathogen interactions (Park et al., 2018).

Here, we discuss the contribution of chloroplasts to plant immunity and their role as a target to pathogen manipulation weakening plant defense. Considering the internal redox environment of the chloroplast and the role of redox regulations in mediating plant responses to stress, we focus on the processes that directly or indirectly depend on chloroplast-associated ROS and redox changes. The role of ROS and redox components in retrograde signaling is not emphasized as it has recently been extensively reviewed (Dietz et al., 2019).

## The PTI/ETI Model of Plant Immunity

The plant immune system relies on patterns-triggered immunity (PTI) and effector-triggered immunity (ETI) which are defined by the recognition mechanism of invading pathogens. PTI, responsible for the non-host-specific resistance, is activated following recognition of pathogen-associated molecular patterns (PAMP) by receptors at the cell surface. PTI initiates defense responses associated with ROS generation, stomata closure, activation of mitogen-activated protein kinases (MAPK) and induction of defense genes. Recognition of the avirulence (*Avr*) genes-coded pathogen effector proteins by cytoplasmic resistance (R) protein receptors triggers ETI which is more robust than PTI and includes the local hypersensitive response (HR) often followed by systemic acquired resistance in the host (Cook et al., 2015). HR is a specialized form of programmed cell death (PCD) characterized by a rapid cell death at the point of pathogen penetration that usually leads to or is linked to resistance associated with Nucleotide Binding Site and Leucine-Rich Repeat domains (NBS–LRR) R-proteins, but is not restricted to the ETI. HR is competent against biotrophs which grow and reproduce in living hosts but it may be beneficial for necrotrophs feeding on dead tissues (Balint-Kurti, 2019).

Oxidative burst associated with a biphasic accumulation of ROS, mainly O_2_^–^ and H_2_O_2_, is a hallmark of plant interactions with incompatible pathogens. The first, non-specific phase of ROS generation is linked to the activity of NADPH oxidase respiratory burst homolog (RBOH) and class III cell wall peroxidases in the apoplast whereas the second one is specifically associated with ETI and occurs in chloroplasts (Shapiguzov et al., 2012). Apoplastic ROS accumulation is sensed in all cellular compartments via different redox-based mechanisms, and ROS produced in the apoplast and in chloroplasts, mitochondria, and peroxisomes are involved in interorganellar communication to trigger the immune response (Mignolet-Spruyt et al., 2016). The oxidative burst originating in different compartments trigger redox-modulated SA signaling with NPR1 (non-expressor of pathogenesis-related gene 1) being the master redox sensor for SA-mediated gene expression in the defense response (Seyfferth and Tsuda, 2014; Herrera-Vásquez et al., 2015).

## Structural Pathways Underlying Chloroplast Signaling

The decrease in the number and size of chloroplasts, the occurrence of plastoglobules and degradation of thylakoids were found markers of biotic stress (Gabara et al., 2012; Zechmann, 2019). In *Botrytis cinerea*-infected plants these changes have been attributed to the accumulation of ROS, especially H_2_O_2_ in chloroplasts (Rossi et al., 2017). Chloroplasts communicate with other organelles via signaling networks and by establishing physical contact with them (Park et al., 2018). Activation of PTI, ETI, and PCD-promoting signals such as H_2_O_2_ and SA, trigger chloroplast re-localization, clustering around the nucleus, and extending stromules, the stroma-filled tubules (Caplan et al., 2015; Ding et al., 2019). A decline in photosynthesis, often accompanying plant immunity, and increase in ROS generation within chloroplasts are likely pre-requisites for stromule formation (Brunkard et al., 2015; de Torres Zabala et al., 2015). During HR, chloroplasts are the major source of H_2_O_2_ which is a defense signaling molecule and induces nuclear gene expression (Yao and Greenberg, 2006). Stromules could facilitate the direct transfer of chloroplast-sourced H_2_O_2_ to the nucleus. Stromule formation increases with enhanced ROS generation in chloroplasts and its frequency is regulated in response to the chloroplast redox status (Brunkard et al., 2015; Caplan et al., 2015; Exposito-Rodriguez et al., 2017). Specific sub-sets of chloroplasts harboring the MSH1 (MUTS HOMOLOG1) protein function as sensory plastids and participate in epigenetic stress memory in plants (Foyer, 2018). *MSH1* silencing results in differential expression of biotic stress-related genes and the function of MSH1 is associated with redox state of these chloroplasts (Virdi et al., 2015).

## The Chloroplastic Electron Transport Chain Carriers and Their Links to Plant Defense

The chloroplast redox state is determined by electron flow through the photosynthetic electron transport chain (PETC) with plastoquinone (PQ) proposed to be the central redox regulator (Potters et al., 2010; Liu and Lu, 2016). PQ is also involved in plant immune response. Light-induced redox changes of the PQ pool regulated HR and defense gene expression (Mühlenbock et al., 2008). PQ content increased in plants treated with pathogen-derived elicitor and acting as an antioxidant, PQ mediated the ROS balance during oxidative stress induced by elicitation (Maciejewska et al., 2002). In *Mesembryanthemum crystallinum*, the PQ redox state modified the response to *B. cinerea* by affecting the activity of antioxidant enzymes and the HR-like response was promoted when the PQ pool was reduced (Nosek et al., 2015). PQ is involved in ABA biosynthesis through the oxidative cleavage of epoxy-carotenoids (Rock and Zeevaart, 1991) and thus linked with the hormone-regulated defense. As PQ reduces O_2_ to O_2_^⋅–^ by semiquinone and O_2_^⋅–^ to H_2_O_2_ by hydroquinone, PQ also regulates defense signaling mediated by these two ROS (Camejo et al., 2016). In *Arabidopsis*, 50 nuclear genes is regulated by the redox state of PQ and the kinases STN7 and CSK1 are involved in this signaling. PQ may also regulate gene expression indirectly through the generation of H_2_O_2_ (Adamiec et al., 2008; Pfannschmidt et al., 2009).

Ferredoxin, the most upstream electron acceptor in PETC, determines the redox status of downstream reductants, e.g., NADPH and thioredoxins. NADPH produced in chloroplasts by ferredoxin-NADP^+^ reductase is used in defense-related anabolic processes and in the regeneration of antioxidants by NADPH-dependent enzymes (Noctor et al., 2006). Ferredoxin and NADPH are involved in redox signaling via ferredoxin-and NADPH-dependent thioredoxin reductases localized in chloroplasts as well as maintenance of redox balance mediated by ascorbate–glutathione cycle. Both processes are implicated in regulating disease resistance (Kuźniak and Skłodowska, 2005; Potters et al., 2010; Hanke and Mulo, 2013). In *Arabidopsis*, NADPH-dependent thioredoxin reductase C (NTRC) working together with H_2_O_2_-reducing 2-Cys peroxiredoxin play a role in innate immunity to non-host *Pseudomonas syringae* pathogens. This redox detoxification system regulates H_2_O_2_ generated in chloroplasts and functions as a negative regulator of disease-associated cell death. The increased susceptibility of *Arabidopsis ntrc* mutant to non-host *P. syringae* correlated with enhanced JA-dependent signaling (Ishiga et al., 2016).

The main leaf ferredoxin, Fd2 is required for resistance against pathogens. Fd2 plays a positive role in PTI-mediated ROS accumulation but negatively regulates the ETI response. The Fd2-knockout mutants exhibited increased susceptibility to virulent *P. syringae* pv. *tomato* DC3000 and the powdery mildew *Golovinomyces cichoracearum*. The Fd2-knockout mutant accumulated more JA following *P. syringae* pv. *tomato* DC3000 infection whereas the SA-mediated defense was compromised (Wang et al., 2018).

Imbalance in PETC may initiate and modulate defense responses, e.g., via ROS-mediated chloroplast-to-nucleus signaling (Karpiński et al., 2013). As activation of immune responses requires redox-mediated transcriptome reprogramming, the ferredoxin-dependent availability of NADPH and redox status of chloroplast thioredoxins may modify chloroplast retrograde signaling and affect nuclear gene expression (Rintamäki et al., 2009). In *Arabidopsis*, 286 nuclear genes were identified to be under the photosynthetic redox control and 76 of these genes encoded products with known functions, e.g., stress response (Fey et al., 2005). Moreover, Fd2 localizes in stromules and therefore it could reduce the redox-regulated transcription factors (TFs), including those required for the expression of SA-dependent genes (Fobert and Després, 2005; Wang et al., 2018).

## Carotenoid, Unsaturated Fatty Acids, and Tocopherol Derived Signaling

In the chloroplast membranes, unsaturated fatty acids, carotenoids, and tocopherols act as ROS quenchers and their oxidation products can regulate defense responses. Tocopherols transfer the stress signals outside the chloroplast, possibly by influencing redox signaling in other organelles. Recently, tocopherols were shown to access endoplasmic reticulum (ER) via hemifused-membranes at plastid-ER contact sites (Mehrshahi et al., 2014). Under stress, changes in the content and composition of tocopherols modulate nuclear gene expression, the profiles of SA, JA, ABA, and ET as well as the formation of defense-related lipid peroxidation products. A link between redox and hormone signaling mediated, respectively, by γ-tocopherol and Ethylene Response Factors (ERFs) which integrates ABA, JA, and ET response to infection, found in vitamin E-deficient *Arabidopsis* mutant, likely represents a mechanism of chloroplast to nucleus retrograde signaling (Müller and Munné-Bosch, 2015; Allu et al., 2017). Altered tocopherol composition in chloroplasts negatively influenced *Arabidopsis* response to *B. cinerea* through enhanced lipid peroxidation and delayed defense activation (Cela et al., 2018).

Apocarotenoids are the products of oxidative cleavage of carotenoids. Interestingly, SA content increase in response to excess light which inhibits ROS accumulation in chloroplasts is dependent on apocarotenoid, β-cyclocitral which interferes with the SA signaling by regulating the localization of NPR1 in the nucleus (Hou et al., 2016).

Pathogens elicit the accumulation of oxylipins which are the products of peroxidation of polyunsaturated fatty acids (PUFA), and those with α,β-unsaturated carbonyls are reactive electrophilic species (RES). The generation of oxylipins in chloroplasts can activate defense signaling and has an impact on gene expression (Farmer and Mueller, 2013). JA originated form PUFA controls gene expression through CORONATINE-INSENSITIVE 1 (COI1), JASMONATE-ZIM DOMAIN (JAZ) proteins, and MYC TFs (Pieterse et al., 2009). RES signal transduction involves the class II TGA TFs and is enhanced by SA, known to inhibit JA signaling. Thus, JA and RES play distinct roles in mediating plant-defense responses (Findling et al., 2018). Moreover, oxylipins identified as signaling molecules, contribute to defense as antimicrobial agents inhibiting pathogen spore germination and growth (Prost et al., 2005).

The production of ^1^O_2_, the predominant ROS in chloroplasts, can increase during pathogenesis favoring oxidative burst. PUFA in thylakoid membranes may act as structural ^1^O_2_ scavengers (Farmer and Mueller, 2013). The protective role of PUFA during pathogenesis was shown in *Arabidopsis* where genetic removal of triunsaturated fatty acids led to increased susceptibility to *B. cinerea* (Mène-Saffrané et al., 2009). Moreover, in *Arabidopsis*–*P. syringae* interaction, massive lipid oxidation is confined to plastid lipids and the HR-related PCD is preceded by ^1^O_2_-dependent lipid peroxidation (Zoeller et al., 2012).

There is compelling evidence that tetrapyrrole signaling contributes to abiotic stress tolerance (Larkin, 2016) but to the best of our knowledge there are no data directly indicating its role in plant–pathogen interactions.

## Chloroplast-Generated ROS in Plant–Pathogen Interactions

Chloroplasts can perceive and propagate PTI signals generated in the apoplast following pathogen recognition. Application of flg22, a conserved peptide of bacterial flagellin, to *Arabidopsis* leaves induces CAS (Calcium-Sensing Sensor)-mediated calcium signaling in the chloroplast stroma. CAS mediated both the basal defense responses (PTI) and HR (ETI) leading to downregulation of photosynthesis-related genes and upregulation of the defensive genes (Nomura et al., 2012). ROS production in chloroplasts was linked to PAMP-induced downregulation of non-photochemical quenching (NPQ) due to weaker accumulation of PSII protein subunits. These PAMP-induced changes in redox balance prime chloroplasts to respond with massive ROS burst upon recognition of effectors during ETI (Göhre et al., 2012). Increased ETI-related ROS contribute to HR-like cell death mediated by MAPK and light-dependent ROS generation in chloroplasts is preceded by inhibition of photosynthetic CO_2_ fixation (Liu et al., 2007; Zurbriggen et al., 2009). Moreover, *Arabidopsis* plants in which H_2_O_2_ generation at PSI was abolished were more susceptible to *P. syringae* pv *tomato* mutant which lacks the ability to secrete effectors, and so only elicits PTI. In this system the pathogenicity of the mutant was rescued (Göhre et al., 2012).

The level of ferredoxin decreases under stress and functional replacement of ferredoxin by a cyanobacterial flavodoxin confer resistance to biotic stress in tobacco. These plants infected with *B. cinerea* showed sustained photosynthetic electron flow, decreased ROS accumulation and enhanced resistance to this necrotroph which invasion is known to be facilitated by HR and oxidative processes mediated by ROS (Govrin and Levine, 2000; Rossi et al., 2017).

ROS-related signaling and the expression of defense require fine-tuning of the prooxidant-antioxidant balance in chloroplasts (Das and Roychoudhury, 2014). Pathogens could activate abiotic stress tolerance mechanisms related to ROS managements to promote virulence and plants defective in these systems and overproducing ROS show increased resistance to pathogens (Sowden et al., 2018). However, the effects depend on ROS amount, timing, and the plant–pathogen interaction. For example, during a non-host interaction of tobacco-*Xanthomonas campestris* pv *vesicatoria*, ROS generated in chloroplasts were essential for the development of HR but not for the induction of pathogenesis-related (PR) genes, and JA and SA accumulation (Zurbriggen et al., 2009). Moreover, the ROS homeostasis is coordinated by NO and the interplay of H_2_O_2_ and NO affects the immune response. The interaction of these redox molecules is required for HR, NO inhibits NADPH oxidase and PCD, and NO-mediated modifications of ascorbate peroxidase and other antioxidant enzymes have regulatory functions under biotic stress (Frederickson Matika and Loake, 2014; Saxena et al., 2016). Moreover, nitrosoglutathione (GSNO), a NO-derived molecule, facilitates the oligomerization of NPR1 through thiol S-nitrosylation (Lindermayr et al., 2010; Corpas et al., 2013).

Infected plants experience episodes of reduced CO_2_ availability to photosynthesis because both foliar pathogenesis and resistance responses can result in stomata closure (Melotto et al., 2008; Grimmer et al., 2012). Consequently, photorespiration is increased and the metabolic integration of chloroplasts with mitochondria and peroxisomes via this pathway contributes to defense (Sørhagen et al., 2013). Photorespiration provides photoprotection by dissipating excess excitation energy in the absence of sufficient CO_2_ as an electron acceptor and reducing ROS generation as well as contributes to redox homeostasis during biotic challenge (Reumann and Corpas, 2010; Eisenhut et al., 2017). Elevated activity of the photorespiratory enzyme, serine:glyoxylate aminotransferase likely confers pathogen resistance in melon by stimulating glycolate oxidase and the intraperoxisomal production of H_2_O_2_, and thereby activating the immune response (Taler et al., 2004). Moreover, H_2_O_2_ originated in chloroplasts and peroxisomes can elicit different responses, and H_2_O_2_ from peroxisomes stimulates stress tolerance whereas that from chloroplasts induces early defense signaling (Sewelam et al., 2014). In *Arabidopsis* overexpressing glycolate oxidase in chloroplasts and the peroxisomal catalase deficient mutant *cat2-2*, producing increased amounts of H_2_O_2_ from the respective organelles, only signals generated by H_2_O_2_ in chloroplasts enhanced resistance to *Colletotrichum higginssianum* (Schmidt et al., 2020).

## Chloroplasts as Targets for Pathogen Manipulation

Pathogens modify chloroplast functions for their benefit. During interactions with hemibiotrophs, this mechanism often relays on suppressing redox-linked SA pathway by activating the antagonistic JA signaling (Robert-Seilaniantz et al., 2011). *Ralstonia solanacearum* uses type III effector proteins called Rips (*Ralstonia*-injected protein) to induce JA accumulation by releasing linolenic acid with its lipase activity. Simultaneously, Rips promote bacterial pathogen growth by suppressing SA and SA-dependent signaling in infected cells (Nakano and Mukaihara, 2018). Coronatine, *P. syringae* phytotoxin with structural similarity to JA which promotes bacterial entry and growth, targets photosynthesis and modulates ROS balance in chloroplasts (Ishiga et al., 2009). The *P. syringae* toxin syringolin and the *Xanthomonas campestris* effector XopJ interfere with the degradation of NPR1 which redox-dependent turnover is required for SA signaling (Büttner, 2016).

*Sclerotinia sclerotiorum* induces stomata opening at the early stages of infection. Oxalic acid secreted by this fungus acidifies the infected tissues, stimulates NPQ by enhancing the conversion of violaxanthin to zeaxanthin and attenuates ROS generation, affecting chloroplast redox status. The dysfunction of the xanthophyll cycle limits ABA biosynthesis by decreasing the violaxanthin precursor for ABA synthesis in chloroplasts. This affects defense responses such as ROS induction and callose deposition which increases plant susceptibility to *Sclerotinia* (Zhou et al., 2015; Zeng et al., 2020).

*P. syringae* effector HopN1 targets the oxygen-evolving complex of PSII, suppresses cell death, attenuates ROS production, callose deposition, and the formation of defense signals in *Arabidopsis* chloroplasts (Rodríguez-Herva et al., 2012). HopI1, the *P. syringae* pv *maculicola* effector localizes to chloroplasts, suppresses SA accumulation, and affects thylakoid stacking (Jelenska et al., 2007). It also recruits cytosolic Hsp70 to chloroplasts suppressing the function of cytosolic Hsp70 in basal defense (Jelenska et al., 2010). Chloroplast proteins identified as targets of virus effectors are components of the PETC (e.g., ferredoxin, Rieske Fe–S), the PSII oxygen-evolving complex and Rubisco, which supports the chloroplast role in plant defense (Bobik and Burch-Smith, 2015).

## Conclusion

The organelle–organelle contacts and inter-compartment communication initiated at the plasma membrane on pathogen recognition are essential for defense. Chloroplasts have emerged as regulatory hubs connecting the primary metabolism and plant defense. They participate in PTI and ETI through ROS/redox systems, retrograde signaling, and phytohormones (Table 1). Chloroplasts are the source and the target of redox regulations which are integrated to the interorganellar signaling network and contribute to the outcome of the plant immune response. Therefore, our integrated understanding of the redox-mediated functions of chloroplasts in photosynthesis and plant immunity will be highly relevant in developing new crops with broad-spectrum resistance to pathogens.

**TABLE 1:** Chloroplast factors involved in plant immune response.

Factor	Role in plant immunity	References
Photosynthesis-derived reactive oxygen species (ROS)	Contribute to pattern-triggered immunity (PTI) and effector triggered immunity (ETI) and chloroplasts are the main source of ROS during hypersensitive response. Chloroplast ROS-mediated retrograde signaling leads to the induction of defense gene expression. ROS generation and signaling in chloroplasts follows ROS bursts in the apoplast triggered by pathogen recognition. The signaling specificity of ROS depends on the chemical characteristics and the ROS-antioxidants balance in chloroplast	Zurbriggen et al., 2009; Göhre et al., 2012; Nomura et al., 2012; Mignolet-Spruyt et al., 2016
Plastoquinone	Regulates defense signaling and gene expression through O_2_^–^ and H_2_O_2_ generation. Mediates ROS balance and affects the activities of antioxidant enzymes. Is linked with abscisic acid (ABA)-regulated defense by contributing to ABA biosynthesis	Rock and Zeevaart, 1991; Maciejewska et al., 2002; Adamiec et al., 2008; Pfannschmidt et al., 2009; Nosek et al., 2015; Camejo et al., 2016
Ferredoxin	Determines the redox status of NADPH and ferredoxin-dependent thioredoxins involved in defense signaling. The increased susceptibility to biotrophic and hemibiotrophic pathogens of ferredoxin-knockout mutants relays on suppressing salicylic acid (SA) pathway and activating the antagonistic jasmonic acid (JA) signaling; Ferredoxin localized in stromules could be involved in redox-mediated transcriptome reprogramming required for activation of immune response	Robert-Seilaniantz et al., 2011; Wang et al., 2018
NADPH	Chloroplast-produced NADPH is involved in redox signaling via NTRC and used in the regeneration of ascorbate and glutathione by NADPH-dependent enzymes in the ascorbate-glutathione cycle which is implicated in regulating disease resistance	Kuźniak and Skłodowska, 2005; Potters et al., 2010; Hanke and Mulo, 2013
NADPH-dependent thioredoxin reductase (NTRC)	The importance of the NTRC in plant immunity is shown by elevated JA signaling and enhanced susceptibility of the *ntrc Arabidopsis* mutant to non-host pathogens	Ishiga et al., 2016
Thioredoxin Trx-h	NtTRXh3 protein localized in chloroplasts is involved in tobacco resistance to viruses by contributing to ROS scavenging and cellular reducing conditions. The redox status of thioredoxins affects nuclear gene expression by modifying chloroplasts retrograde signaling	Rintamäki et al., 2009; Sun et al., 2010
Tocopherols	Involved in the antioxidant protection of chloroplast membranes and in the transfer of stress signals outside the chloroplast via plastid- endoplasmic reticulum contact sites Tocopherols content and composition modulate nuclear gene expression, the profiles of defense hormones and PUFA-derived defense products	Mehrshahi et al., 2014; Müller and Munné-Bosch, 2015; Allu et al., 2017; Cela et al., 2018
Apocarotenoids	Chloroplast-generated signaling molecules produced by carotenoid cleavage link chloroplast activity and nuclear gene expression. They interfere with SA signaling by regulating the localization of NPR1, a redox-sensitive transcription co-activator, in the nucleus	Bobik and Burch-Smith, 2015; Brunkard et al., 2015; Hou et al., 2016
Polyunsaturated fatty acids (PUFA)	Biosynthetic precursors of JA which is central to modulating defense against necrotrophs, participates in systemic acquired resistance and usually antagonizes SA-mediated defense Independently of being the precursors of JA, PUFA are sinks for ROS in chloroplasts	Mène-Saffrané et al., 2009; Farmer and Mueller, 2013
Oxylipins	Reactive electrophilic species signaling molecules interfering with TGA transcription factors-mediated SA pathway which also exhibit antimicrobial activity	Prost et al., 2005; Findling et al., 2018
Calcium sensor protein (CAS)	Thylakoid-localized calcium-binding protein which connects chloroplasts to immune responses triggered during PTI and ETI and regulates the biosynthesis of SA via the chloroplast isochorismate pathway. CAS is involved in PAMP-induced defense gene expression, including SA biosynthesis genes, through ^1^O_2_-mediated retrograde signaling. SA generally mediates defense against biotrophic/hemibiotrophic pathogens and systemic acquired resistance	Nomura et al., 2012; Bobik and Burch-Smith, 2015

## Author Contributions

EK and TK took responsibility for the integrity of the work as a whole. Both authors contributed to the article and approved the submitted version.

## Conflict of Interest

The authors declare that the research was conducted in the absence of any commercial or financial relationships that could be construed as a potential conflict of interest.
